# Advanced hydrogel mesh platform with neural stem cells and human umbilical vein endothelial cells for enhanced axonal regeneration

**DOI:** 10.1063/5.0244057

**Published:** 2025-04-01

**Authors:** Jong-Tae Kim, Sung Woo Han, Dong Hyuk Youn, Harry Jung, Eun-Ho Lee, Sung-Min Kang, Yong-Jun Cho, Jin Pyeong Jeon

**Affiliations:** 1 Institute of New Frontier Research, Hallym University College of Medicine, Chuncheon 24252, Republic of Korea; 2 Department of Green Chemical Engineering, Sangmyung University, Cheonan 31066, Republic of Korea; 3 Department of Neurosurgery, Hallym University College of Medicine, Chuncheon 24252, Republic of Korea

## Abstract

One of the major obstacles to neural recovery following intracerebral hemorrhage (ICH) is the cavity-like lesion that occurs at the site of the hemorrhage, which impedes axonal regeneration. Here, we aim to address this challenge by investigating the migratory mechanisms of neural stem cells (NSCs) within the cavity *in vitro* using a hydrogel and endothelial cells. Mouse NSCs (mNSCs) isolated from the subventricular and subgranular zones using the 3D hydrogel culture were evaluated for their neurogenic, extracellular matrix (ECM), and adhesion-related mRNA expression compared to microglia (BV2) and secretory factors of human umbilical vein endothelial cells (HUVECs) *in vitro* and in *vivo* conditions. A hydrogel mesh combining mNSCs and HUVECs was developed for its therapeutic potential. mNSCs exhibit high stemness, neurogenesis, and ECM remodeling capabilities. mNSCs demonstrated close interaction with HUVECs and the surrounding vascular structures in *in vitro* and *in vivo* studies. Furthermore, mNSCs could degrade high concentrations of fibrin to facilitate migration and adhesion. mNSCs and HUVECs formed mesh networks through cell–cell contacts and maintained the structure through Matrigel support, potentially ensuring sufficient survival and regeneration capabilities. Our proposed hydrogel mesh platform with mNSCs and HUVECs demonstrated successful maintenance of cell survival and provision of structural support for the delivered cells by promoting ECM remodeling and neurogenesis, which may aid in axonal regeneration in the cavity lesions following ICH.

## INTRODUCTION

Although the initial surgical removal of intracerebral hemorrhage (ICH) in the acute phase is successful, the subsequent development of a cavity at the injury site can impede neuroregeneration and functional recovery. A range of stem cell therapies have been explored to address cavity repair resulting from extensive cell loss and inflammatory responses following damage.^1–3^ One promising approach is the transplantation of exogenous neural stem cells (NSCs) into the injured site, where the cells may survive, migrate, and differentiate into neurons, thus promoting neurological recovery.^4^ However, the survival rate of injected NSCs at the graft site was only less than 5% from 2 weeks to 2 months post-hemorrhage,^5,6^ a limitation that must be addressed for their practical clinical application.

With advancements in tissue engineering technologies, various types of neural structures and cells have been investigated to mimic the heterogeneous nature of neural tissues.^7–9^ Since fabricating neural grafts may replace damaged neural cells, it is crucial to provide adequate mechanical stability, microenvironment, and architecture to enhance the survival capabilities of transplanted NSCs while promoting axonal regeneration and connections with grafted cells in injured sites.^9^ Suspended cells detaching from culture plates lack cell-to-cell interactions and cell-to-extracellular matrix integration; therefore, providing suitable substrates is critical for successful treatment. In addition to structural support, supplying extracellular matrix (ECM) can offer extrinsic signals that influence the fate of NSCs. For instance, NSCs under soft ECM conditions of 0.1–0.5 kPa appear to facilitate neuronal differentiation and cell migration, whereas conditions over >1 kPa tend to promote glial cell differentiation.^10–12^ These findings underscore the importance of interactions between grafted cells and ECM in enhancing cell survival and differentiation capabilities. Therefore, proper control of ECM lysis may be a potential intervention target for NSCs transplanted into injured brain sites forming cavities.

In the central nervous system (CNS), the success of transplanted NSCs and their differentiation depends on the formation and function of vascular structures. The vasculature in the CNS provides oxygen and nutrients during development and maintains homeostasis.^13^ Although sprouting angiogenesis continuously remodels and expands the vascular network in developing or healthy adult brains, CNS vasculature remains in a quiescent state, as only a few endothelial cells proliferate.^14,15^ Currently, achieving rapid and successful vascularization for transplanted cells *in vivo* is a significant challenge in the fields of tissue engineering and regenerative medicine.^16^ Therefore, introducing exogenous endothelial cells or stimulating existing vascular endothelial cells could potentially enhance rapid vascularization and neural regeneration.

Previously, we reported an isolation procedure for NSCs from the subventricular zone (SVZ) and the subgranular zone (SGZ) of the hippocampus in the mouse brain using a hydrogel-based three-dimensional (3D) culture system comprised of collagen/fibrin.^17^ However, the fundamental mechanism by which NSCs migrate from brain tissues to hydrogels has not yet been investigated. We observed continuous migration of NSCs via guidance cells, which underscores the need for further research on these phenomena. Here, we evaluated regeneration-associated cells, potentially possessing capabilities for proliferation, migration, and differentiation by providing a blood clot-like environment *in vitro* to mimic an injured brain. Furthermore, we sought to identify the ECM regulatory mechanisms involving both NSCs and endothelial cells that play a crucial role in the regeneration process. Through these efforts, we aimed to elucidate the endogenous regenerative mechanism of the injured brain and propose a therapeutic potential for cavity repair using cellular engineered hydrogels with NSCs and human umbilical vein endothelial cells (HUVECs) for neuronal regeneration following central nervous system (CNS) injury.

## RESULTS

### Hydrogel culture and isolation of mouse neural stem cells

**FIG. 1.**
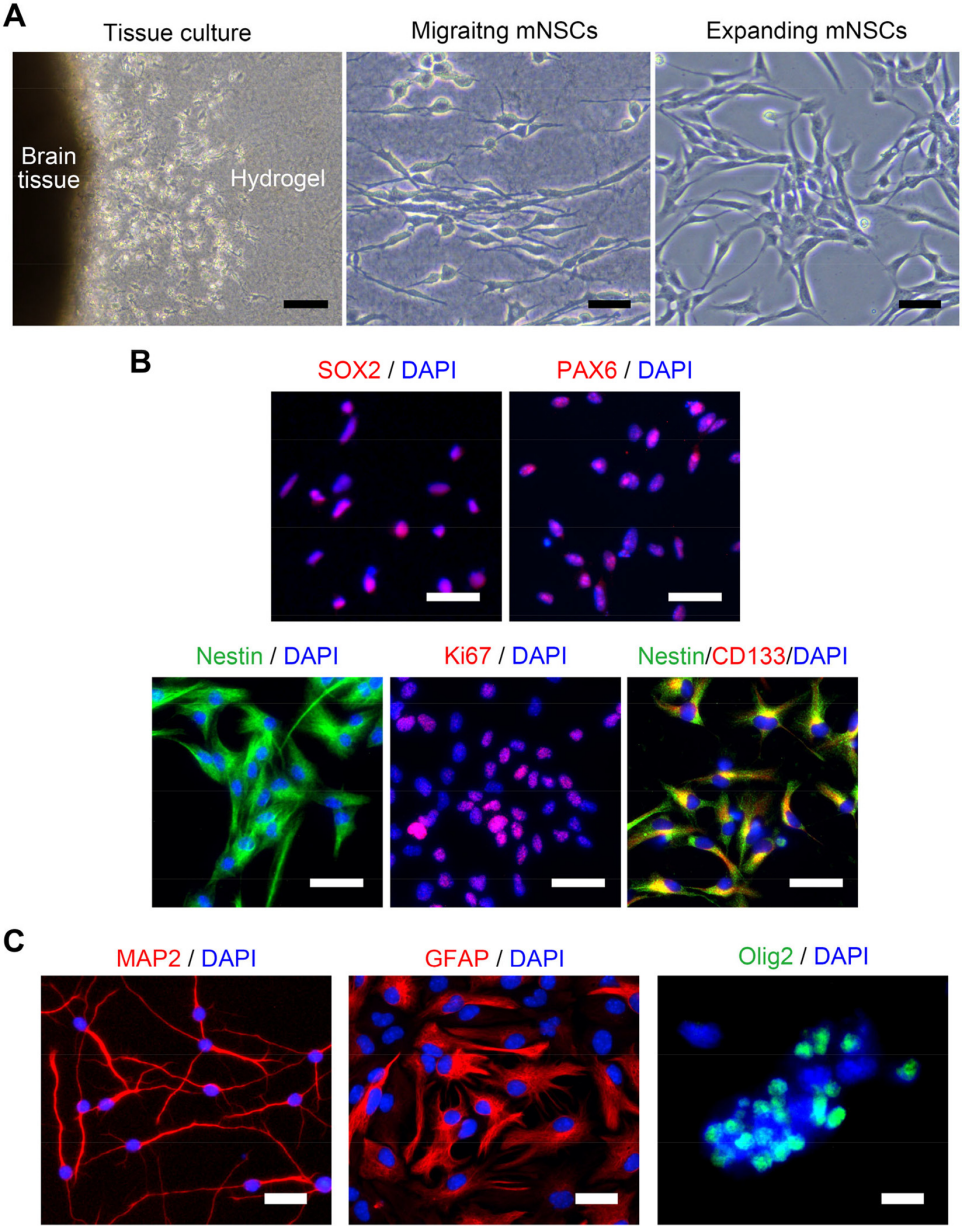
Neural stem cell isolation and characteristics. (a) Representative images show outgrowth of neural stem cells (NSCs) in a 3D brain tissue culture system. The left and middle panels illustrate migration of NSCs from brain tissue to hydrogel and their morphologies. The right panel reveals expanded NSCs on a Matrigel-coated culture plate in a conventional culture method after hydrogel lysis. Scale bars are 200 and 50 *μ*m. (b) Isolated NSCs express specific NSC markers: SOX2, PAX6, and Nestin, along with enhanced proliferation markers such as Ki67 and CD133. Scale bars are 50 *μ*m. (c) The differentiation abilities of NSCs into neurons, astrocytes, and oligodendrocytes are demonstrated. Scale bars are 20 *μ*m.

We hypothesized that mNSCs from stem cell niches in the brain could be induced to migrate toward the cavity by providing suitable ECM conditions, such as collagen/fibrin hydrogels, after brain injury. Cells migrating from the SVZ and SGZ to the hydrogels were observed and proliferated consistently over 3 weeks. The cells were isolated selectively and expanded successfully [Fig. 1(a)]. To determine whether the migrating cells were NSCs of mice, additional staining with NSC proliferation markers was performed. Cell-surface markers characteristic of NSC or neural progenitor cells were expressed [e.g., SOX2, PAX6, nestin, Ki67, and CD133; Fig. 1(b)], but not markers related to fibroblasts or macrophages. Accordingly, these migrating cells were identified as mNSCs. The mNSCs also exhibited the potential for multi-lineage differentiation into neurons, astrocytes, and oligodendrocytes [Fig. 1(c)].^17^

### Gene expression of mNSCs

**FIG. 2.**
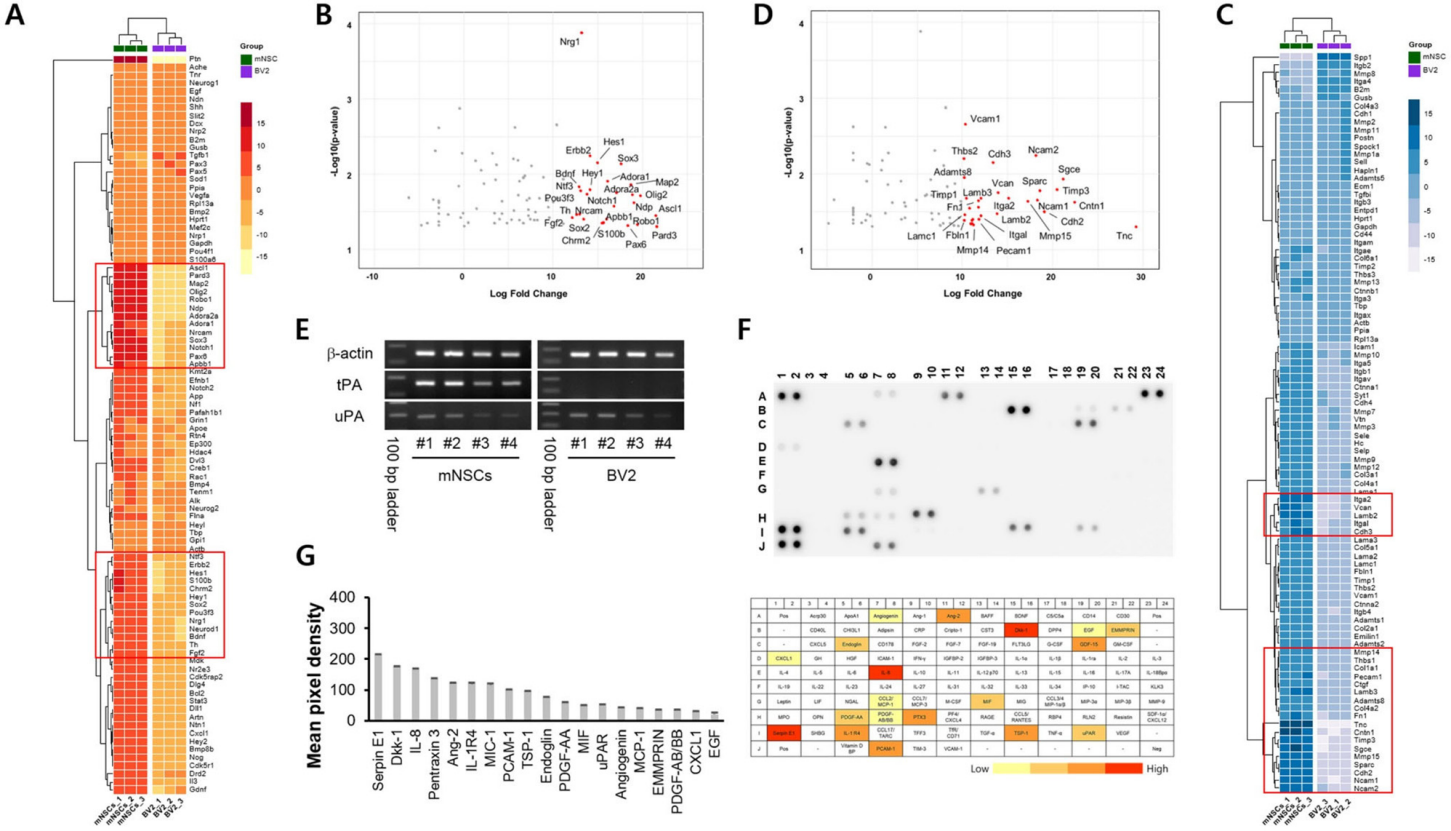
Gene expression of mNSCs and cytokine profiling of HUVEC. (a) Heatmap of 92 genes, including housekeeping genes, depicts the neurogenesis-associated mRNA expression in mNSC compared to BV2 cells. (b) A scatterplot indicates a greater than 12-fold difference in neurogenesis-related values between mNSCs and BV2 cells, with red spots emphasized. (c) Heatmap of 92 genes, including housekeeping genes, illustrates the mRNA expression associated with ECM and adhesion molecules in mNSCs compared to BV2 cells. (d) A scatterplot displays a more than 10-fold difference in ECM and adhesion molecule values between mNSCs and BV2 cells with red spots emphasized. (e) mRNA expressions of tPA and uPA in mNSCs and BV2 batches. (f) and (g) Cytokine expression profile of HUVEC to investigate the potential effects on ECM secretion.

Next, we compared the gene expression of isolated mNSCs with that of the BV2 cell line to assess whether any microglial cells might have been unintentionally included during the isolation process. This comparison was made to address concerns regarding potential microglia contamination. To further define the specific properties of mNSCs, we conducted a qPCR screening to investigate potential mechanisms of regeneration and cell migration, using primer sequences for genes associated with neurogenesis and extracellular matrix and adhesion molecules. Transcription factors, such as PAX6, SOX2, and SOX3, crucial for the maintenance of neural stem cells and the regulation of neurogenesis, were highly expressed in mNSCs [Figs. 2(a) and 2(b)]. Additionally, genes related to cell survival, neurotrophic factors, transcription regulation, neuronal differentiation, and metabolic processes were also highly expressed (e.g., Ascl1, Hes1, Robo1, Notch1, and BDNF).

Given the significant migration ability of mNSCs, gene expressions targeting ECM and adhesion molecules were evaluated. mNSCs demonstrated increased mRNA expressions of tenascin C (TNC), versican (Vcan), fibronectin (Fn1), vitronectin (Vtn), fibulin 1 (Fbln 1), and laminin subunits, which are important factors for NSC adhesion, survival, and differentiation.^18,19^ Genes related to neural cell and vascular cell adhesion molecules were also prominently expressed {e.g., Ncam1, Ncam2, Vcam1, and cadherins [Figs. 2(c) and 2(d)]}. Furthermore, we explored the potential fibrinolytic properties of mNSCs by analyzing the expression of tissue plasminogen activator (tPA) and urokinase plasminogen activator (uPA) [Fig. 2(e)]. The results indicated that mNSC expressed mRNA for tPA and uPA [Fig. 2(e)], with tPA being particularly highly expressed in mNSC compared to BV2.

### Cytokine expression of HUVEC

To assess the cytokine effect potentially related to mNSC, we analyzed secretions from HUVEC culture media. Under normal conditions, the serine protease inhibitor clade E member 1 (SERPINE1), an endothelial plasminogen activator inhibitor, was highly expressed. Interestingly, the urokinase-type plasminogen activator receptor (uPAR) also showed lower expression levels under the same conditions in HUVEC [Figs. 2(f) and 2(g)].

### Evaluation of gene delivery

**FIG. 3.**
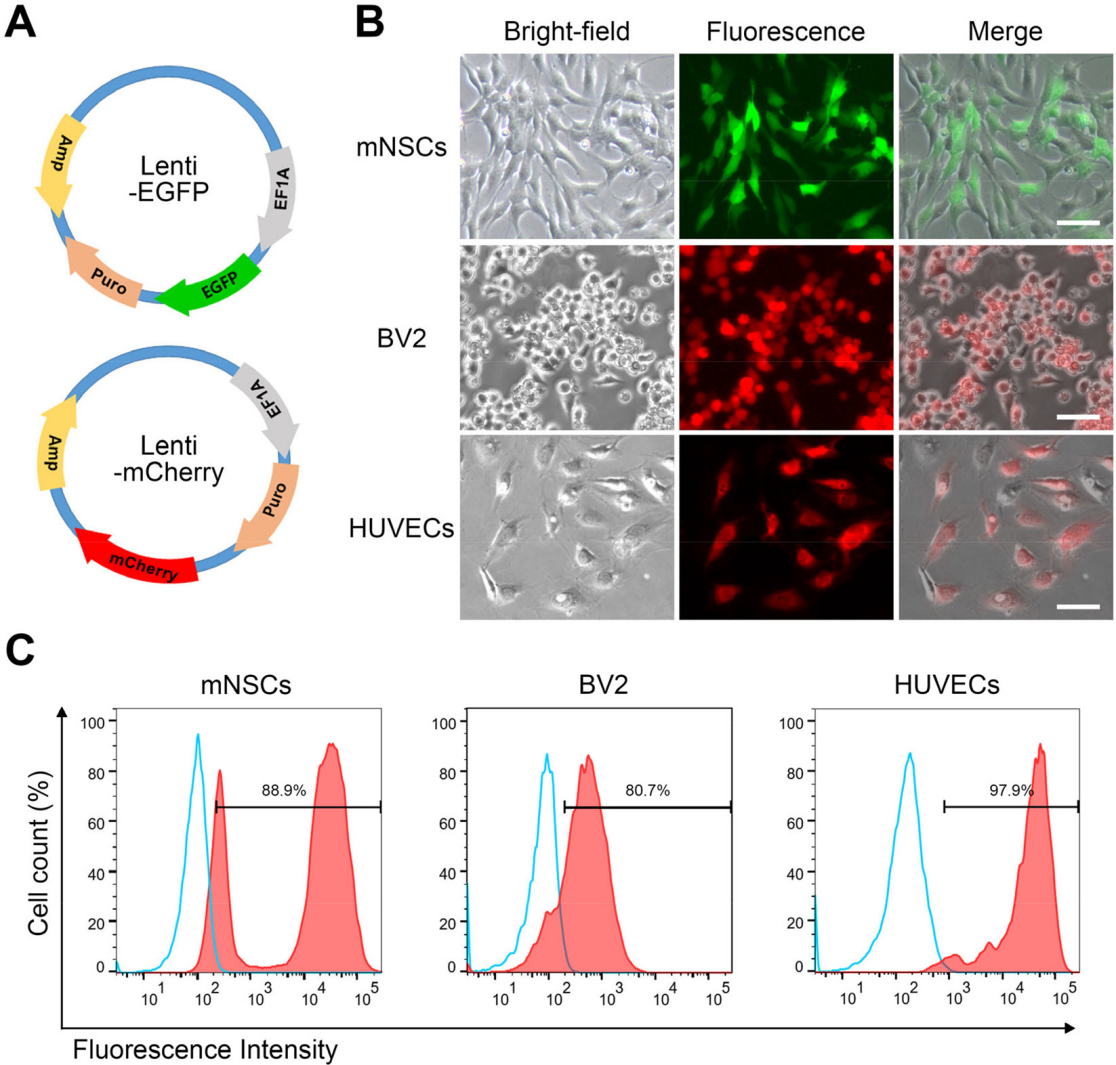
Transduction of GFP and mCherry into mNSCs, BV2, and HUVECs. (a) Representative images depict the vector designs used for lentiviral transduction of EGFP and mCherry. (b) Fluorescence microscopy of living cells showing EGFP expression in mNSCs and mCherry expression in BV2 and HUVEC cells, respectively. Scale bars measure 100 *μ*m. (c) Quantitative analysis of fluorescence intensities in individual cells using flow cytometry. The blue histogram represents the isotype control and the red histogram indicates positive cells. The percentage reflects gated population.

To investigate the specific behavior of mNSCs, cells used in these experiments were transduced with fluorescence reporter genes using a lentiviral gene delivery system [Fig. 3(a)]. After gene delivery, EGFP was stably expressed in mNSCs, with an expression rate of 88.9% [Figs. 3(b) and 3(c)]. mCherry was also stably expressed in BV2 and HUVEC, with expression rates of 80.7% and 97.9%, respectively. The cells were continuously passaged without any morphological changes.

### Specific relationship between mNSCs and HUVEC

**FIG. 4.**
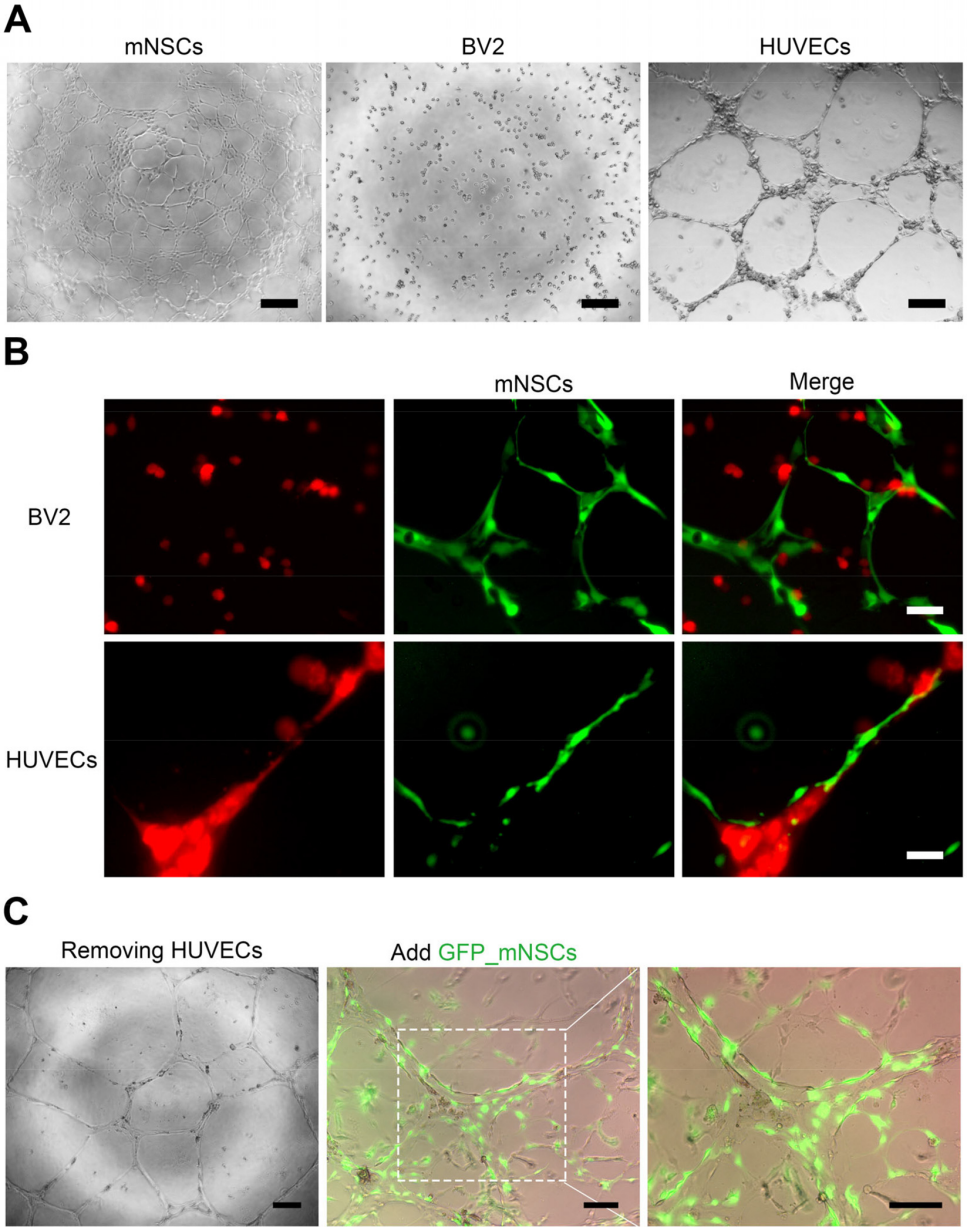
*In vitro* assay of mNSCs in co-culture system with BV2 and HUVEC. (a) Representative images show cell morphologies and adhesions on Matrigel prior to co-culture use. Scale bars measure 200 *μ*m. (b) Integration and migration of mNSCs within a HUVEC co-culture environment, not seen in BV2 cells. Scale bars measure 50 *μ*m. (c) mNSCs demonstrate high affinity for the vascular-like structure from which HUVEC were removed. Scale bars are 200 and 100 *μ*m.

We studied the behaviors of mNSCs by using ECM and vasculature mimics provided by Matrigel and HUVEC, and explored potential migration mechanisms of mNSC. The mNSCs were observed on Matrigel bedding, exhibiting migration and the formation of cell-to-cell networks from the first day. HUVEC formed vascular-like network structures on the Matrigel bed, while BV2 displayed a random distribution [Fig. 4(a)]. We then assessed whether the mNSCs were incorporated into the vascular-like structures of HUVEC during this migration. Upon introducing EGFP-labeled mNSCs into the pre-formed vascular structure of the mCherry-labeled HUVEC on the Matrigel bed, the EGFP-mNSCs selectively engaged with the vascular-like structure, showing a preference for the mCherry-HUVEC over the BV2 [Fig. 4(b)]. Interestingly, even in the vascular-like structures from which HUVEC had been removed, the EGFP-mNSCs aligned along the remnants of the structure and connected to each other [Fig. 4(c)].

### Proteolytic potency and flexibility of mNSCs

**FIG. 5.**
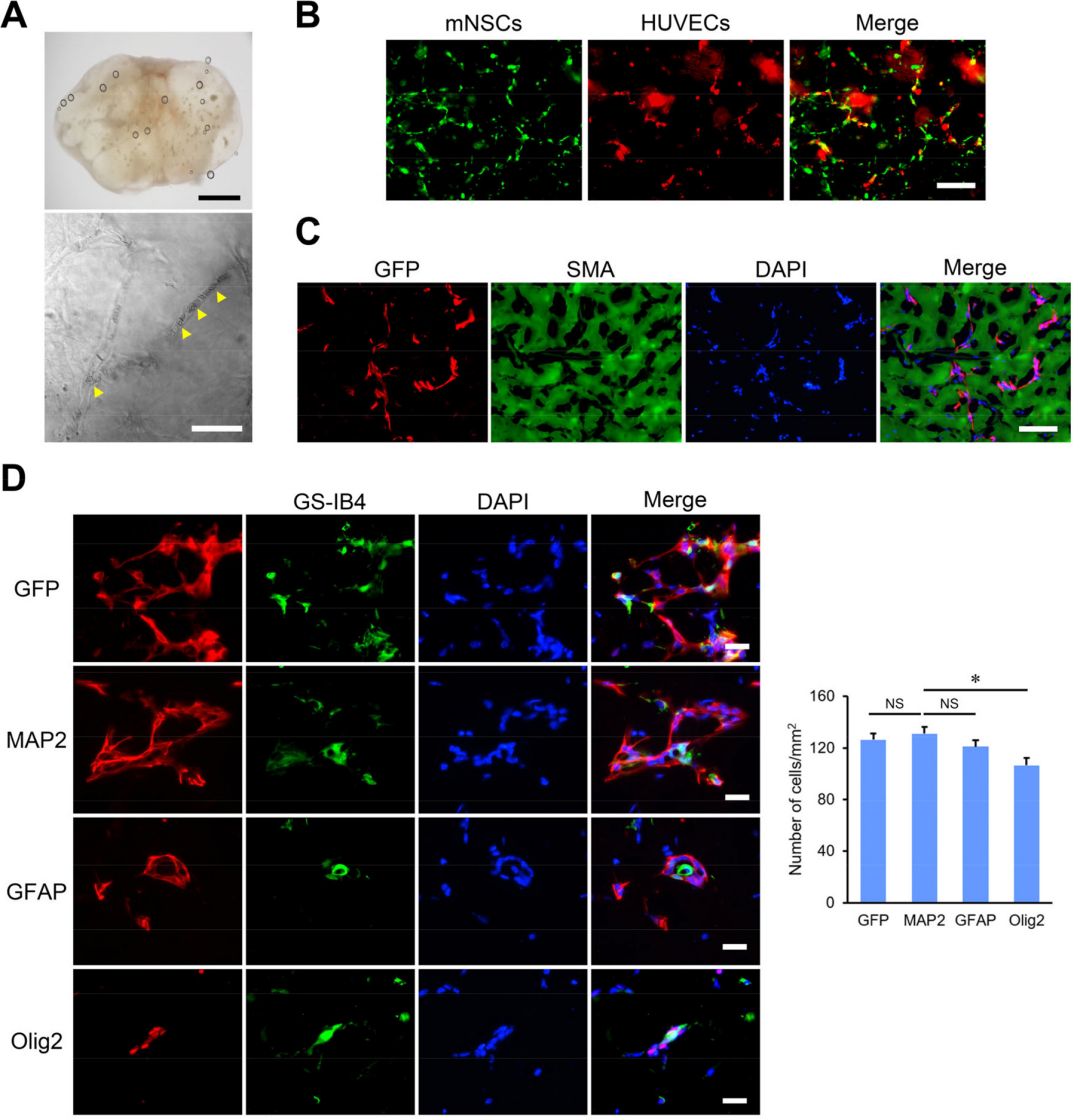
*In vivo* evaluation of migration and interaction between mNSCs and HUVECs using the Matrigel plug assay. (a) Representative images display blood vessels infiltrating the Matrigel plug. The yellow arrowheads indicate red blood cells perfused through the vessels at 7 days post subcutaneous injection. Scale bars are 1 mm and 50 *μ*m. (b) Unstained fluorescence images illustrate the survival of mNSCs and HUVECs with expressed GFP and mCherry signal in the Matrigel plug before fixation. Scale bar is 200 *μ*m. (c) Immunostaining reveals SMA-positive fibroblasts that do not overlap with mNSCs and ECM degradation. Scale bar is 100 *μ*m. (d) Representative images depict interactions between mNSCs and endothelial structures, potentially differentiating into neurons, astrocytes, and oligodendrocytes. Scale bars are 25 *μ*m. Error bars, SEM; NS, not significant. ^*^P < 0.05.

To investigate the interactions between mNSCs and HUVEC under *in vivo* conditions, a Matrigel plug assay was conducted. GFP-mNSC/mCherry-HUVEC with Matrigel was injected subcutaneously to evaluate its characteristics regarding glycoproteins and proteoglycans, which are critical in scar formation post-brain injury.^20,21^ One week later, blood vessels formed within the Matrigel plug, and the perfusion of red blood cells (RBCs) was observed in the vascular structure [Fig. 5(a)]. mNSCs and HUVEC survived and continuously expressed fluorescence within the Matrigel plug [Fig. 5(b)]. Immunofluorescence staining indicated that mNSCs did not differentiate into SMA-positive fibroblasts. The absence of fluorescence signals in vacant spaces indirectly suggests that mNSCs could utilize the adjacent ECM [Fig. 5(c)]. mNSCs were positioned close to the structures of GS-IB4 positive cells, likely representing HUVEC or vascular endothelial cells derived from the host mouse. Furthermore, mNSCs differentiated into neurons, astrocytes, and oligodendrocytes within the Matrigel plug without external signals driving the differentiation [Fig. 5(d)].

### Development of a cellular engineered hydrogel mesh

Rapid vascular ingrowth within engineered hydrogels is essential for addressing insufficient blood perfusion in implants. We hypothesized that fabricating a mesh-like hydrogel using PDMS micro-pillars with mNSCs and HUVECs could promote a neuronal microstructure with rapid vascularization in a large surface area. A mesh structure would also be effective for oxygen and nutrient supply because oxygen and nutrient supply from peripheral blood vessels to the surrounding tissues occurs within approximately 200 *μ*m.

**FIG. 6.**
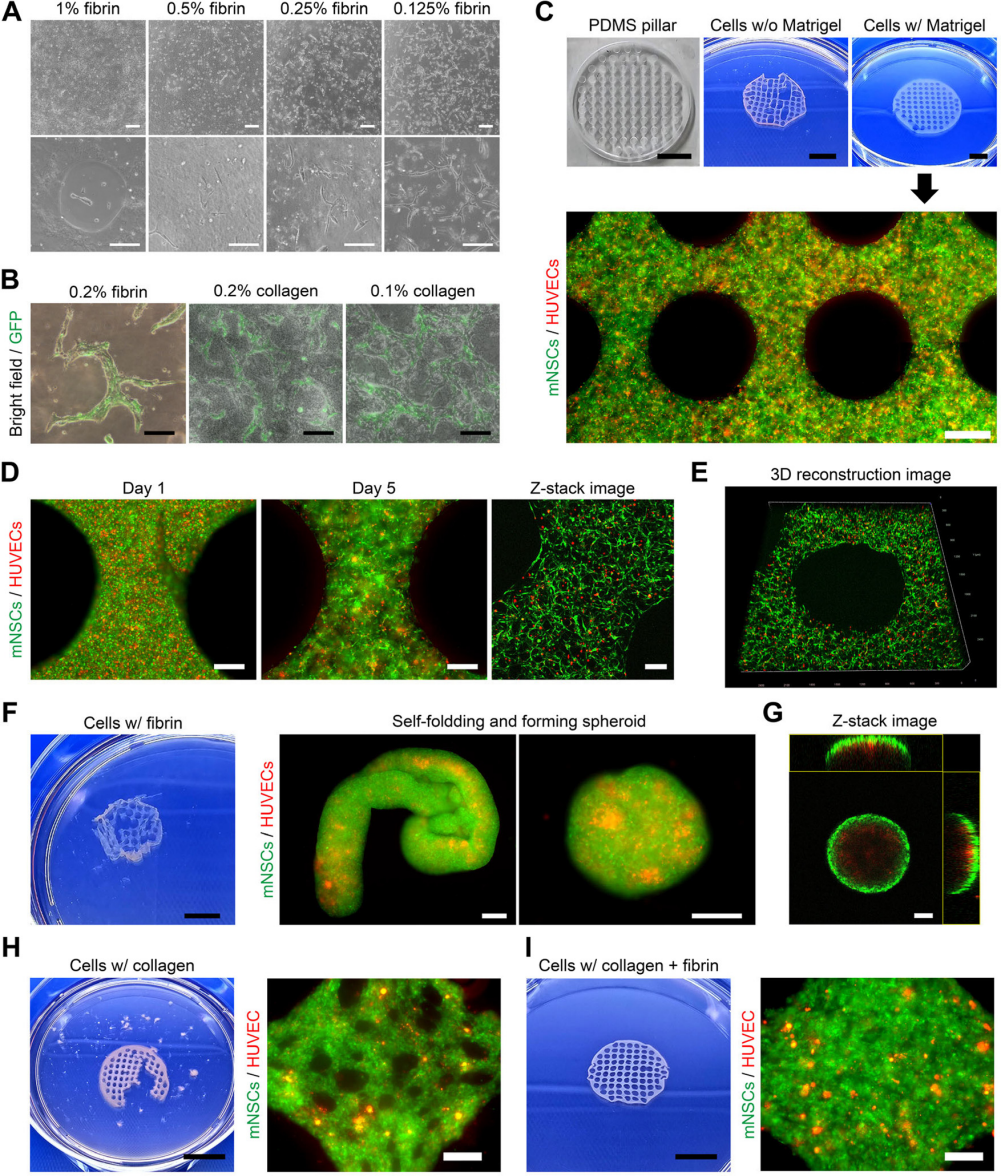
*In vitro* comparison of spreading and migration abilities of mNSCs and the fabrication of a cellular engineered hydrogel mesh. (a) Representative images display cell morphologies varying with fibrin concentration after 3 days of culture under orbital shaking conditions. Scale bars are 200 *μ*m. (b) Images showing mNSCs forming chains for migration in culture conditions of 0.2% fibrin and collagen hydrogel. Scale bars are 100 *μ*m. (c) Construction of a cellular engineered hydrogel mesh using PDMS-based micro-pillar with GFP-mNSCs and mCherry-HUVECs. Fluorescence images illustrating that mNSCs exhibit network-like structures similar to neuronal networks. Scale bars are 10 mm and 500 *μ*m. (d) and (e) Confocal microscope images reveal the spatial distribution of GFP-mNSCs and mCherry-HUVECs and the 3D reconstruction within the hydrogel mesh. Scale bars are 200 *μ*m. (f) Macro-image displays an unstable fibrin mesh where the ECM was degraded by mNSCs. GFP-mNSCs and mCherry-HUVECs in the degraded ECM were divided into several parts and aggregated into self-folding and spheroidal shapes. Scale bars are 10 mm and 200 *μ*m. (g) Orthogonal imaging from different planes of the confocal microscope. Scale bar is 100 *μ*m. (h) Macroscopic images showing breakdown of the 0.25% collagen hydrogel due to its weak physical properties. Fluorescence image shows degraded collagen ECM in the hydrogel. Scale bars are 10 mm and 200 *μ*m. (i) Although the structure of the hydrogel mesh was preserved with a combination of 0.25% collagen and 0.5% fibrin in the macroscopic image, the matrix of the hydrogel mesh was almost degraded, indicating rapid shrinkage of the mesh size because it was mainly maintained by intercellular binding. Scale bars are 10 mm and 200 *μ*m.

To examine whether a cellular engineered hydrogel mesh can facilitate neurogenesis with vascularization, various natural hydrogels were tested to develop a transferrable mesh structure using PDMS-based micro-pillars. Since mNSCs specifically expressed tPA [Fig. 2(e)], their degradable capability was confirmed at different concentrations of fibrin similar to CNS injuries such as ICH. It was observed that mNSCs could degrade 1% of highly concentrated fibrin and exhibited high migratory capacity based on the concentration [Fig. 6(a)] after 3 days of culture. mNSCs also demonstrated chain-forming migration in culture conditions of 0.2% fibrin and collagen hydrogel [Fig. 6(b)], suggesting that mNSCs could migrate into the injured area with fibrin degradation of the hemorrhage lesion.

We investigated the safe delivery of mNSCs and HUVECs in a scar-forming environment using a fabricated hydrogel mesh composed of Matrigel on PDMS-based micro-pillars containing mNSCs and HUVECs. Matrigel is known to support cell attachment, survival, growth, differentiation, migration, and tissue vascularization.^22^ Additionally, it has the property of forming a three-dimensional gel at temperatures above 10 °C.^23^ We hypothesized that by using Matrigel to create a hydrogel mesh, we could form a 3D gel with evenly distributed cells. mNSCs and HUVECs formed networks via cell–cell contacts, maintaining the structure within the Matrigel support [Fig. 6(c)]. Both cell types were evenly distributed by day 1, and mNSCs successfully organized three-dimensional neural networks around HUVECs within 5 days [Fig. 6(d) and 6(e)]. In the presence of 0.5% fibrin mesh, the fibrin hydrogel degraded quickly, retaining features of cell–cell aggregation, such as self-folding and spheroidal shapes (supplementary material Fig. S2). Furthermore, mNSCs seemed to encapsulate HUVECs, possibly indicating adequate survival and regenerative capabilities [Figs. 6(f) and 6(g)]. Additionally, we evaluated properties of hydrogel meshes based on their compositions using 0.25% collagen and a 0.25% collagen/0.5% fibrin combination. As expected, the hydrogel mesh made with 0.25% collagen and mixed cells did not maintain its physical structure. It degraded slowly. Although the structure of the hydrogel mesh was preserved with the 0.25% collagen and 0.5% fibrin combination, the matrix of the hydrogel mesh was almost degraded, indicating rapid shrinkage of the mesh size because it was mainly maintained by intercellular binding [Figs. 6(h) and 6(i) and supplementary material Fig. S2].

## DISCUSSION

Although the therapeutic effects of NSCs in CNS injury have been continuously reported, specific markers for treatment have not yet been designated due to the inherent plasticity of NSCs, complicating their clinical use. Through this study, we identified characteristics of mNSCs such as neurogenesis and ECM-associated adhesion mRNA expression, along with their potential relationships with endothelial cells. Isolated mNSCs from the SVZ and SGZ were found to express essential NSC markers such as SOX2, PAX6, and nestin, which are crucial for self-renewal, pluripotency, and neurogenesis. Furthermore, they displayed a proliferation marker (Ki67) and a maintenance marker (CD133). SOX2 and PAX6 are recognized as NSC markers and play interactive roles in gene regulation during neuronal development.^24^ Additionally, SOX3, as depicted in Fig. 2(a) and 2(b) images, indicative of adult neuroprogenitor cells, is engaged in sustaining telencephalic neurogenesis.^25^ CD133 is involved in various cellular processes,^26^ including PI3K/AKT,^27^ Wnt/β-catenin,^28,29^ Smad,^30^ and the Hedgehog signaling pathway.^31^ These signaling pathways are crucial for the self-renewal, proliferation, migration, survival, differentiation, and axonal regeneration of NSCs. Moreover, the soft matrix in 3D models can regulate CD133 mRNA levels, which are linked to pluripotent genes such as SOX2, OCT4, and Nanog.^32–34^ Therefore, mNSCs derived from our culture platform can be utilized to explore the properties of human NSCs suitable for therapeutic applications.^35,36^ Brain parenchyma outside the niche is known to limit the neurogenic potential of transplanted NSCs or neuroblasts. ECM plays an integral role in the stem cell niche as a dynamic and complex environmental factor that regulates cell behaviors.^37^ Consequently, it is crucial for transplanted cells to effectively regulate ECM stiffness for migration to the hematoma cavity in the brain, particularly in ICH. In this study, we probed mRNA expressions of ECM and adhesion molecules in mNSCs to ascertain their potential impact on plasticity and capacity for ECM remodeling. NSC niches in adult mouse brains displayed high ECM-specific mRNA expressions of Tnc and Contactin-1/F3 (Cntn1), which are implicated in the migration of NSCs or neuroblasts.^38^ Cntn1 mediates neuron–glial contacts by interacting with ECM components such as Tnc and Tnr. Additionally, it engages the glial surface receptor RPTPβ/phosphacan, which is critical for neuronal migration and regulates the process formation of newborn cortical neurons by modulating the RhoA signaling pathway.^39^ Given that Timp1 and metalloproteinase (MMP) genes are involved in ECM remodeling, mNSCs could effectively migrate to injured sites to support neurogenesis by regulating the ECM's remodeling and stiffness.^40,41^ Our findings align with prior studies that describe niche-specific ECM and NSC markers.^38^ In specific cases of ICH, it is essential for cells to regenerate in the area previously occupied by the blood clot, which subsequently transforms into a cavity. Based on this, we hypothesized that transplanted mNSCs could dissolve the stiff blood clots characteristic of ICH and fill the resulting cavity gaps. To validate our hypothesis, we investigated the potential fibrinolytic properties of mNSCs and BV2 by analyzing tPA and uPA. Unlike BV2, which expresses only uPA, mNSCs expressed both tPA and uPA. tPA is responsible for the degradation of fibrin by activating plasminogen to plasmin, while uPA activates cell-associated plasminogen during cell migration or tissue remodeling in non-proteolytic conditions.^42^ In correlation with HUVEC's secretory cytokine analysis [Figs. 2(f) and 2(g)], HUVEC exhibited high expression of endothelial plasminogen activator inhibitor (a serine protease inhibitor, serpin E1), functioning as plasminogen activator inhibitor 1 (PAI-1).^43^ In post-stroke conditions, migration of neuroblasts to the injured area closely associated with blood vessels and astrocytes.^44–46^ To directly determine the role of endothelial cells in mNSC migration, we performed *in vitro* co-culture and *in vivo* experiments similar to Figs. 4 and 5. Although the *in vivo* experiment did not show transplanted cells in the injured brain, transplanted HUVECs and host endothelial cells within hydrogel directed the migration and differentiation of mNSCs into neurons. Based on these findings, we suggest that combining NSCs with vascular endothelial cells may be more effective in restoring brain function after ICH through ECM remodeling and fibrin degradation compared to the mere administration of NSCs. Additionally, vascular structures could serve as a migratory scaffold for mNSCs at the injured sites requiring regeneration.^47,48^

**FIG. 7.**
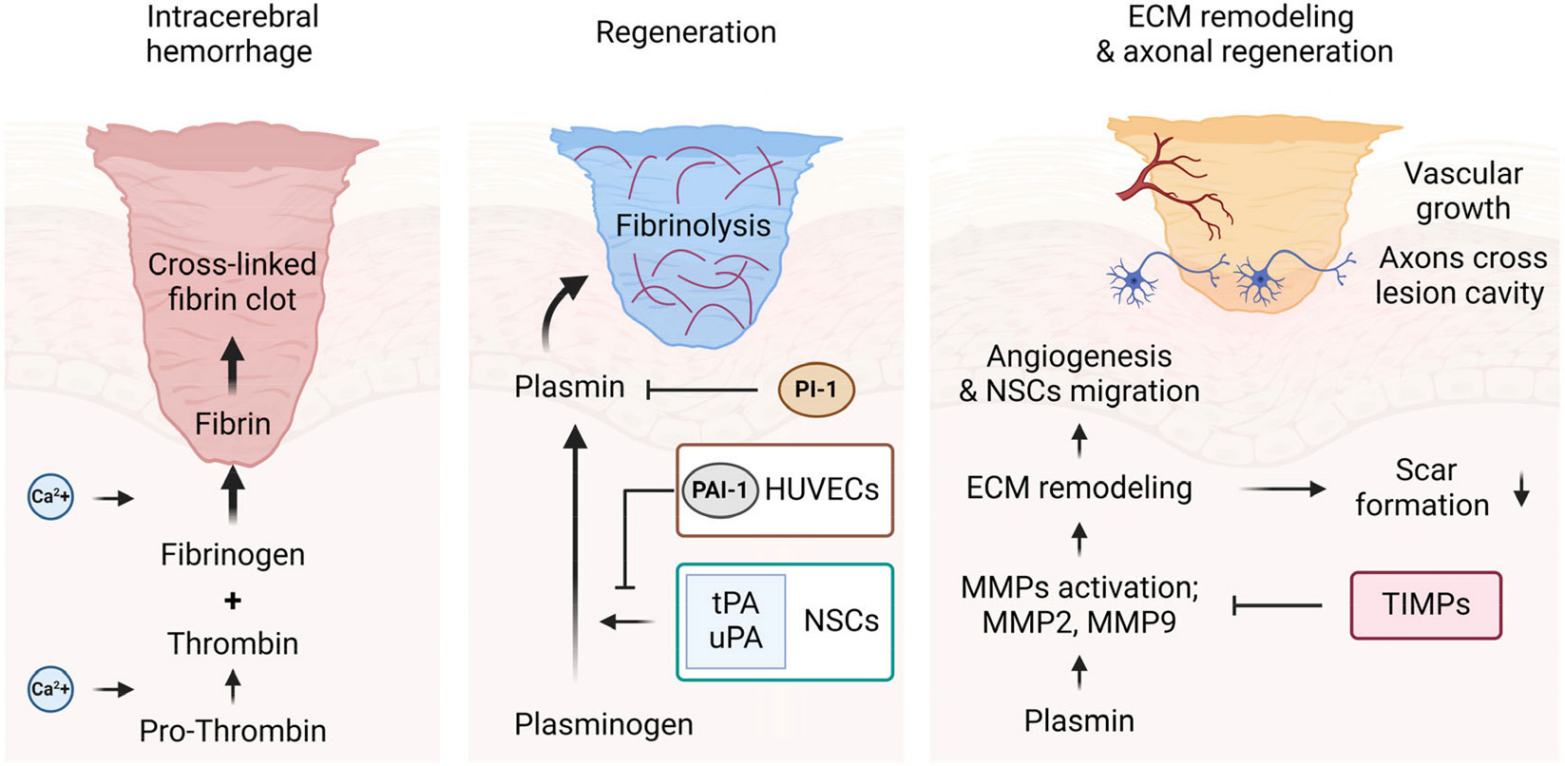
Graphical representation of a mechanism underlying the study conclusion. A cellular engineered hydrogel mesh with mNSCs and HUVECs can be used as a large-scale carrier to fill the cavity in injured brain tissues after ICH. The transplanted cells could initiate regeneration, promoting ECM remodeling and NSC migration, which fosters angiogenesis and neuronal regeneration within the lesion cavity.

Here, we generated a cellular engineered hydrogel mesh using natural ECM hydrogels for several reasons. First, hydrogels exhibit matrix plasticity that enhances both cell–matrix and cell–cell adhesions by promoting stable integrin binding with focal adhesion kinase in cells.^49^ This property is particularly advantageous in 3D brain tissue cultures, where it supports the proliferation and external outgrowth of mNSCs via chain-migration.^17^ The hydrogel ECM also creates cellular environments that promote vascular assembly, invasion, and migration of endothelial cells. Regarding the survival and engraftment of transplanted cells, including NSCs and endothelial cells, intensive hydrogel-based delivery, rather than single cells, can significantly enhance therapeutic effects.^17,50^ Second, while synthetic hydrogels offer the advantage of easily tunable mechanical properties by adjusting the concentration, cross-linking degree, and solidification time to simulate desired stiffness, they pose significant clinical challenges. These include concerns over biological safety, potential inflammatory responses, and cytotoxicity associated with the cross-linking process, which often entails the use of reagents or ultraviolet rays.^51^ Third, rapid vascular ingrowth within engineered hydrogels is crucial for overcoming the issue of insufficient blood perfusion in implants.^52^ We hypothesized that fabricating a hydrogel mesh with mNSCs and HUVECs could ensure a neuronal microstructure through rapid and functional vascularization by providing a large surface area.^53,54^ Our findings indicate that mNSCs and HUVECs interact through distinct mechanisms that influence their migratory behaviors. Understanding these interactions is essential for developing new therapeutic strategies for brain injuries, particularly ICH. To address the challenges of stable cell delivery and neuronal regeneration, we propose that pre-fabricated cellular engineered hydrogel meshes can serve as large-scale carriers to bridge gaps in damaged brain tissue (Fig. 7).

There are some limitations to this study. First, we used Matrigel to simulate the ECM environment of a scar. However, its use in clinical practice may be limited due to ECM being secreted by Engelbreth–Holm–Swarm (EHS) mouse sarcoma cells. Second, the actual regeneration process unfolds very slowly, making the integration of transplanted blood vessels with the host's blood vessel structure and their long-term stability a potential challenge. Third, despite successful transplantation, concerns about immunogenicity and biocompatibility may persist when used *in vivo*. Risks of immune responses or rejection could limit the clinical applicability of NSCs or endothelial cells. Therefore, further research is necessary to optimize hydrogel properties, enhance vascularization, and improve long-term outcomes of the engineered hydrogel mesh in brain tissue repair.

## CONCLUSION

We directly replicated previous findings that mNSCs can migrate through blood vessels in the brain after injury. We showed that chains of migrating mNSCs depended on ECM and endothelial cells through a bidirectional relationship *in vitro*. We demonstrated that our model could recapitulate complex interactions required for brain repair and regeneration. In particular, the promotion of ECM remodeling and neurogenesis at injured sites where bleeding occurs could be achieved using a cellular engineered hydrogel mesh with mNSCs and HUVECs. Based on our research findings, further optimization and fine-tuning of the cellular engineered hydrogel mesh in experimental and clinical settings is necessary for treating a variety of brain injuries in actual clinical practice.

## METHODS

### Mouse neural stem cell culture

Isolation and culture of mouse neural stem cells (mNSC) were performed with reference to previous reports.^17^ Briefly, brain tissues from C57BL/6J mice were surgically extracted and minced into 1-mm pieces. These minced tissues were then encapsulated in a 0.1% collagen (MS-BIO, Korea) and fibrin/thrombin (Sigma-Aldrich, Korea) solution to form a gel, treated with Rho kinase inhibitor (Y-27632, Stemcell Technologies, Vancouver, BC, Canada) to achieve stabilization for the initial three days, and maintained under a 3D shaking-culture condition for three weeks at 37 °C in a 5% CO_2_ incubator. The growth medium consisted of 1× N2 supplement (Gibco, Thermo Fisher Scientific, Waltham, MA, USA), 1× B27 supplement (Gibco), 1× GlutaMAX (Gibco), 20 ng/ml EGF (Peprotech, Korea), 20 ng/ml bFGF (Peprotech), and 10 *μ*g/ml gentamicin in a 1:1 (vol/vol) mixture of Neurobasal media (Gibco) and Dulbecco's Modified Eagle's Medium/nutrient mixture F-12 (DMEM/F12) (Welgene, Korea). Subsequently, cells proliferating in the collagen/fibrin hydrogel were collected using enzymatic dissociation with 0.05% type I collagenase (Worthington Biochemical, Lakewood, NJ, USA) treatment and further expanded on Matrigel (Corning Inc., Corning, NY, USA)-coated plates using conventional culture methods with 0.05% trypsin/EDTA (Sigma-Aldrich, Korea).

### Differentiation of mouse neural stem cells

Cells were cultured on Matrigel-coated 4-well chamber slides (SPL Life Sciences, Korea) with specific differentiation media for 2 weeks to assess the differentiation potential of mNSC. Neuronal differentiation was initiated using a medium comprising neurobasal medium, 1× B27 supplement (Gibco), 1× GlutaMAX (Gibco), 1× CultureOne Supplement (Gibco), and 200 *μ*M ascorbic acid (Sigma-Aldrich). Astrocyte differentiation was induced using DMEM high glucose, 1× N2 supplement (Gibco), and 1% FBS (Gibco). For oligodendrocyte differentiation, the medium included neurobasal medium, 1× B27 supplement (Gibco), 5 *μ*g/ml N- acetyl-L-cysteine (Sigma-Aldrich), 30 ng/ml 3, 3, 5-triiodo-L-thyronine (Sigma-Aldrich), 2 ng/ml brain-derived neurotrophic factor (Peprotech), and 2 ng/ml ciliary neurotrophic factor (Peprotech). Upon differentiation completion, cells underwent fixation and subsequent immunofluorescence staining for analysis.

### Cell lines used *in vitro*

HUVEC was supplied by ATCC (Cat no. CRL-1730, Manassas, VA, USA) and BV2 cells were supplied by Cytion (Cat no. 305156, Eppelheim, Germany). HUVECs were cultured with endothelial cell growth medium (PromoCell, Heidelberg, Germany). The medium was changed every 3 days. Cells were placed in a 5% CO_2_ incubator using general culture methods. BV2 was cultured with DMEM high glucose (Welgene, Korea) supplemented with 10% FBS in a 5% CO_2_ incubator using a general culture method.

### Production and concentration of lentivirus

Virus production followed established protocols.^17,55^ 293 T cells, at approximately 90% confluence in T75 flasks, were transfected using Lipofectamine 3000 (Invitrogen, Carlsbad, CA, USA). The employed lentiviral packaging plasmids included 4.5 *μ*g of pMD2.G and 6.5 *μ*g of psPAX2 (gifts from Didier Trono; Addgene #12259 and #12260). Lentiviral expression plasmids consisted of 11.5 *μ*g of mCherry (VB201011-1032fvd), custom-cloned by Vector Builder (VectorBuilder Inc, Chicago, IL, USA), or 11.5 *μ*g of pEGIP (a gift from Linzhao Cheng; Addgene #26777). Supernatants were collected every other day for 3 days and filtered using a 0.45 *μ*m pore size syringe filter. Concentration of lentivirus was then performed via ultracentrifugation at 25 000 rpm at 4 °C for 2 h. The virus pellet was resuspended in 500 *μ*l of serum-free media, aliquoted, and stored at −80 °C until use.

### Lentiviral transduction

Each cell was seeded at 1 × 10^6^ cells in a T75 flask to transduce either EGFP for mNSC or mCherry for HUVEC and BV2 cells. The following day, the cells underwent transduction at the required dilution using lentiviral EGFP or mCherry with 5 *μ*g/ml polybrene (Santa Cruz, CA, USA) in the respective growth media. After a 24-h transduction period, the medium was refreshed with growth media and cultures were expanded using conventional methods. After expansion culture, fluorescence emission was recorded by fluorescence microscope and flow cytometry (FACS Canto II, BD Biosciences, San Jose, CA, USA).

### *In vitro* co-culture assays

To examine the interaction between mNSC and HUVEC or BV2, 200 *μ*l of Matrigel (Corning) was added to a 24-well plate and allowed to gel for 20 min at 37 °C in an incubator. Subsequently, 2 × 10^4^ cells of mCherry-expressing HUVECs or BV2 cells were seeded on the Matrigel. The following day, 1 × 10^4^ cells of GFP-expressing mNSC were co-cultured with the HUVEC or BV2 layers. The next day, fluorescence emission was recorded for GFP (excitation wavelength 488 nm) and mCherry (excitation wavelength 561 nm) using a laser confocal microscope (LSM 710, Carl Zeiss, Jena, Germany).

### Immunostaining

Cells were fixed with 4% paraformaldehyde for 20 min at room temperature and rinsed three times with 1× PBS. Antigen blocking was performed using 5% normal goat serum with 0.1% Triton X-100 (Sigma-Aldrich) in 1× PBS for 30 min at room temperature. Cells were incubated with primary antibodies overnight at 4 °C; these included rabbit anti-SOX2 (1:100, Novus Biologicals, Littleton, CO, USA), rabbit anti-PAX6 (1:100, Abcam, Cambridge, UK), mouse anti-nestin (1:50, Santa Cruz), rabbit anti-Ki67 (1:200, Abcam), rabbit anti-CD133 (1:200, Cell Signaling Technology, Inc.), rabbit anti-MAP2 (1:1000, Abcam), rabbit anti-glial fibrillary acidic protein (GFAP) (1:1000, Abcam), rabbit anti-Olig2 (1:500, Novus Biologicals), mouse anti-SMA (1:50, Santa Cruz), and Alexa fluor 488 conjugated Griffonia simplicifolia isolectin B4 (GS-IB4). Following this, cells were rinsed three times with 0.05% tween 20 (Sigma-Aldrich) in 1× PBS and incubated with secondary antibody using goat anti-mouse Alexa 488, goat anti-rabbit Alexa 488 or Alexa 594 antibodies (Jackson ImmunoResearch Laboratories, Inc., West Grove, PA, USA). Finally, cells were counterstained for 10 min with 10 *μ*g/ml of 4′,6-diamidino-2-phenylindole dihydrochloride (DAPI) in 1× PBS, and mounted with ProLong Gold antifade reagent (Invitrogen, Carlsbad, CA, USA).

### Screening with real-time quantitative polymerase chain reaction (qPCR)

Total RNA was extracted using TRIzol reagent (Invitrogen) following the manufacturer's protocols. The RNA concentration was assessed by spectrophotometer (Eppendorf Bio-Spectrometer kinetic, Hamburg, Germany), and 1 *μ*g of RNA was reverse transcribed using the Maxime RT PreMix Kit (iNtRON Biotechnology, Seoul, Korea). Screening of target genes was performed by real-time quantitative PCR (qPCR) using the Power SYBR^®^ Green PCR Reagents Kit (Applied Biosystems, Foster City, CA, USA). PCR amplification was carried out with gene-specific primers for the neurogenesis and extracellular matrix and adhesion molecules using the Accutarget™ qPCR screening kit (SM-0000-20 for mouse origin, Bioneer, Korea) as follows: 1 cycle of pre-denaturation at 95 °C for 10 min, 40 cycles of denaturation at 95 °C for 15 s, annealing at 58 °C for 20 s, and elongation at 72 °C for 30 s. Target gene expression levels were normalized with endogenous GAPDH, and calculated using the comparative cycle time method with qPCRsoft 4.1 software on a qTOWER 3G (Analytik Jena, Jena, Germany). Genetic screening was visualized using the heatmap (version 1.0.12) and ggplot (version 3.5.1) in R studio package with the adjusted parameters p ≤ 0.05 and −log2foldchange ≥ 10. RT-PCR was performed to determine tPA and uPA mRNA expression levels of mNSCs and BV2 using primers from supplementary material Table 1.

### Profiling of cytokine secretion

HUVEC cells were cultured in T75 flasks until they reached 90% confluency, using endothelial cell growth medium (ECGM, PromoCell, Heidelberg, Germany). The culture medium was then replaced with fresh 10 ml. Supernatants collected on day 3 were centrifuged for 15 min at 2000 × g to remove cell debris. 500 *μ*l of the supernatant was immediately used for the Proteome Profiler Human XL Cytokine Array Kit assay (R&D Systems Inc., Minneapolis, MN, USA) according to the manufacturer's guidelines. Cytokine secretion from HUVEC was quantified using optical density measurements by the ImageQuant LAS 500 (GE Healthcare Bio-Sciences Corp., Piscataway, NJ, USA) and Image J software (version v1.54j).

### *In vivo* Matrigel plug assay

GFP-mNSC and mCherry-HUVEC were mixed in a 1:1 ratio and suspended in a final 2 × 10^6^ cells/50 *μ*l volume of pre-chilled Matrigel. The cells were transferred into a pre-chilled 1/2 ml 27 G-needle syringe (Becton Dickinson, Franklin Lakes, NJ, USA) and kept in an ice bucket until used. After inducing anesthesia with 2% isoflurane and O_2_, the posterior flank of C57BL/6 mice was exposed and the hair trimmed. The cell-suspended Matrigel was then injected subcutaneously and allowed to gel. Cyclosporine A was administered at 10 mg/kg for one week. Subsequently, the Matrigel plugs were removed and cryosectioned into 10 *μ*m thick sections.

### Cellular engineered hydrogel mesh

To ensure efficient oxygen and nutrient exchange, the spacing between micro-pillars of the hydrogel mesh was controlled to 500 *μ*m in diameter after fabrication. This diameter is generally considered the diffusion limit for peripheral blood vessels within tissues.

To construct a cellular engineered hydrogel mesh comprising mNSCs and HUVEC, a micro-pillar master mold was fabricated using polydimethylsiloxane (PDMS) (Sylgard 184, Dow Corning, Inc.). The acrylic master mold was first created by machining acrylic plates with a computer numerical control (CNC) milling machine (TOOLI-34H, David Motion Technology, Korea). The micro-pillar structure was designed using 3D CAD software (Inventor 2023, Autodesk Inventor Fusion, Autodesk Inc., USA) and programmed into the CNC mill. Then, the acrylic plate with the designed structure was drilled using a flat end mill with a diameter of 1 mm (1REM 010 040 S06, JJTOOLS, Korea). Subsequently, to create the PDMS micro-pillar mold, PDMS was thoroughly mixed with Sylgard 184 silicone elastomer and curing agent in a 10:1 (w/w) ratio, and air bubbles were evacuated in a vacuum desiccator at room temperature. The PDMS mixture was poured onto the acrylic master mold, cured overnight at 60 °C, and then the micro-pillar mold was separated from the acrylic master mold (supplementary material Fig. S1).^53^ The PDMS micro-pillar master mold was then autoclaved using a sterile pouch, dried, and exposed to UV for 30 min in biological safety cabinets prior to usage. A cellular networked mesh was formed from 5 × 10^6^ cells of GFP-mNSCs and mCherry-HUVEC at a 1:1 ratio, suspended in either 900 *μ*l of pre-chilled Matrigel, 0.5% fibrin, 0.25% collagen, or 0.25% collagen/0.5% fibrin combination, and introduced into the micro-pillar mold on a 100 mm culture dish. The hydrogels were solidified for 30 min at a 37 °C incubator, supplemented with a culture media mixture containing mNSCs and ECGM, and cultured for 5 days under orbital shaking conditions with media exchanges every other day. Cell dispersion and migration within the hydrogel mesh were analyzed using a confocal microscope.

### Statistical analysis

All data are presented as mean values ± the standard error of the mean (SEM). One-way ANOVA using Tukey's *post hoc* test was predominantly utilized for statistical analysis unless specified otherwise. P-values are denoted by asterisks as follows: ^*^p < 0.05.

## SUPPLEMENTARY MATERIAL

See the supplementary material for the structure and details of the PDMS-based micro-pillar mold (see supplementary material Fig. S1); the mechanical stability characterization of the cell network between hydrogel conditions using fibrin, collagen, and collagen/fibrin combination (see supplementary material Fig. S2); and the list of primers for RT-PCR (see supplementary material Table I).

## Data Availability

The data that support the findings of this study are available from the corresponding author upon reasonable request.
